# Development and validation of a method for human papillomavirus genotyping based on molecular beacon probes

**DOI:** 10.1371/journal.pone.0207930

**Published:** 2018-11-29

**Authors:** Jiangfeng Lyu, Yuefeng Yu, Caixia Pan, Jing Zhou, Xuyi Ren

**Affiliations:** Research and Development Centre, Hangzhou DiAn Medical Laboratory, Hangzhou, Zhejiang, China; University of Helsinki, FINLAND

## Abstract

We describe a new assaying system for the detection and genotyping of human papillomavirus (HPV) based on linear-after-the-exponential-PCR(**L**ATE-PCR) and melting curve analysis. The 23 most prevalent HPV strains (types 6, 11, 16, 18, 31, 33, 35, 39, 42, 45, 51, 52, 53, 56, 58, 59, 66, 68, 70, 73, 81, 82, and 83) are assayed in two sealed reaction tubes within 2 h. Good sensitivity and specificity was evaluated by testing cloned HPV DNA and clinical samples. The detection limit was 5–500 copies/reaction depending on the genotype. No cross-reactivity was observed with the other HPV types that are not covered by our method or pathogens tested which were commonly found in female genital tract. When compared with the HPV GenoArray Diagnostic kit, the results from 1104 clinical samples suggest good overall agreement between the two methods,(98.37%, 95% CI: 97.44%–98.97%) and the kappa value was 0.954. Overall, this new HPV genotyping assay system presents a simple, rapid, universally applicable, sensitive, and highly specific detection methodology that should be useful for HPV detection and genotyping, therefore, is potentially of great value in clinical application.

## Introduction

Cervical cancer is one of the most prevalent cancers in women worldwide, with estimated 527,624 new cases and 265,672 deaths in 2012. Over 85% cervical cases were diagnosised in developing countries [1]. Cytological screening—the Papanicolaou (Pap) test—has greatly reduced the incidence of invasive cervical cancer over the past 50 years [2]. However, Pap test has limited diagnosis sensitivity, which leads to missed diagnosis of grade II, grade III cervical intraepithelial neoplasia (CIN) and even cervical cancer[3]. The majority of cervical cancer cases are caused by persistent infection with HPV, particularly with high-risk genotypes (HPV16, 18, 31, 33, 35, 39, 45, 51, 52, 56, 58, 59, 66, and 68) [1, 4, 5]. Molecular assays for the detection of HPV DNA in cervical specimens and have been developed and widely used in clinics. These molecular methods are more sensitive than the Pap test for the detection of CIN II and CIN III [6, 7]. Currently, the most commonly used HPV detection and genotyping method in China is the GenoArray test (HPV GenoArray test kit; Hybribio Ltd, Hong Kong). However, the limitations of these assays are that they are prone to contamination and are time consuming. In addition, the diagnosis of borderline cases is difficult because of the read-outs being solely based on direct visualization [8].

In this study, we developed a new assay that uses LATE-**P**CR and **M**elting curve analysis (LPM) for the DNA detection and genotyping of the 23 most common HPV strains (types 6, 11,16, 18, 31, 33, 35, 39, 42, 45, 51, 52, 53, 56, 58, 59, 66, 68, 70, 73, 81, 82, and 83) [9–10]. We evaluated this new method based on its clinical performance and concluded that it is faster and more convenient than the current commercially available kits.

## Materials and methods

### Sample collection

One liquid-based cytology (LBC) sample was collected from each of 1108 patients aged 19–89 years (median age, 43 years) who visited the DiAn Medical Laboratory for routine HPV screening or genotyping from August to December 2017. Additional 920 HPV carrying samples (40 samples for each HPV type including HPV types 6, 11, 16, 18, 31, 33, 35, 39, 42, 45, 51, 52, 53, 56, 58, 59, 66, 68, 70, 73, 81, 82, and 83), which diagnosised positive using the HPV GenoArray test kit or by type-specific PCR sequencing (for HPV types 70, 73, 82, and 83), were selected and stored at −80°C until use. The chosen samples were collected from 920 patients aged 23–92 years (median age, 45 years) from January 2016 to May 2017. All samples were collected using a cervical cytobrush and stored in PreservCyt solution (U.S. Cyt Company)tubes along with the cytobrush. The cervical sample collection and followup studies were approved by the ethics committee of DiAn Medical Laboratory (Reference number DA-YXLC-20170619-1).

### DNA extraction

The LBC samples were used for genomic DNA extraction using a QIAamp DNA Mini kit (Qiagen, Hilden, Germany) as per manufacturer’s instruction. DNA was eluted from the columns with 100 μL AE buffer and stored at −20°C.

### Primers and molecular beacon probes design

Various consensus primers have been reported for the amplification of a specific fragment of L1 the region of HPV DNA, including GP5+/6+, MGP, MY09/11, and PGMY09/11 [11–13]. After considering amplification efficiency and the need for molecular beacon designation, GP6+/MY11 were chosen as the LATE-PCR primer set to generate the single strand DNA template for hybridization of the corresponding molecular beacon probe. The design of molecular beacon probes were aided by the DNA folding program mfold (http://unafold.rna.albany.edu/?q=DINAMelt/Quickfold) and the probe–target hybrid folding program DINAMelt (http://unafold.rna.albany.edu/?q=DINAMelt/Two-state-melting) was used to predict the possible hybrid structures and T_m_ values. Sample quality is monitored by amplifying a 100-base pair (bp) fragment of the glyceraldehyde 3-phosphate dehydrogenase (GAPDH) gene using the primers G100f 5′-CTGCTCACATATTCTGGAGGAG-3′ (forward) and G100r 5′- AAAAGCAGCCCTGGTGACC-3′ (reverse). All molecular beacon probe sequences and corresponding targets are listed in Table 1.

**Table 1:** Molecular beacon probes used in LATE-PCR and their corresponding hybridized target sequences.

Probe	Nucleotide Sequence 5′→3′
P6	FAM-CCGGC ACGCAGTACCAACATGACACTATGTGCATC GCCGG-DAB
HPV6	ACGCAGTACCAACATGACATTATGTGCATC
HPV11	ACGCAGTACAAATATGACACTATGTGCATC
HPV16	ACGCAGTACAAATATGTCATTATGTGCTGC
P58	HEX-CCGGC TCGTAGCACTAACATGACATTATGCACTGAAG GCCGG-DAB
HPV58	TCGTAGCACTAATATGACATTATGCACTGAAG
HPV52	TCGTAGCACTAACATGACTTTATGTGCTGAGG
HPV33	TCGCAGTACTAATATGACTTTATGCACACAAG
P68	HEX-CCCCG TACCACTCCCAGGACCAATTTAACTTT CGGGG-DAB
HPV68	TACCACTCGCAGTACCAATTTTACTTT
HPV18	TACCACTCGCAGTACCAATTTAACAAT
P59	CY5-CCGGC CGTAGTACCAATCTGTCTGTGTGTGCTTC GCCGG-DAB
HPV31	CGTAGTACCAATATGTCTGTTTGTGCTGC
HPV59	CGCAGCACCAATCTTTCTGTGTGTGCTCT
HPV35	CGTAGTACAAATATGTCTGTGTGTTCTGC
HPV42	CGTAGTACTAACATGACTTTGTGTGCCAC
P51	FAM-CCGGC TAGAAGTACTAATTTGACTATTAGCACTGCTGCTGCTCAG GCCGG-DAB
HPV51	TAGAAGTACAAATTTAACTATTAGCACTGCCACTGCTGCG
HPV56	TAGAAGTACTAACATGACTATTAGTACTGCTACAGAACAG
HPV81	CAGAAGCACCAATTTTACTATTTGCACAGCTACATCTGCT
MM4	TAGAAGTACCAATTTAACCATTAGCACTGCTGTTACTCAA
P66	FAM-CCCGC ACCAACATGACTATTAATGCAGCTAAAAGC GCGGG-DAB
HPV66	ACCAACATGACTATTAATGCAGCTAAAAGC
P83X	HEX-CCCCG AGTACCAATATTACTATTACAGCTGCTAC CGGGG-DAB
HPV83	AGTACCAATATTACTATTTCAGCTGCTGC
HPV84	AGCACCAATTTTACTATTAGTGCTGCTAC
P73	HEX-CCCGC AAGCACTAATTTTTCTGTATGTGTAGGTACA GCGGG-DAB
HPV73	AAGCACTAATTTTTCTGTATGTGTAGGTACA
P45	CY5-CCGC GTACTAACTTTACATTATGTGCCT GCGG-DAB
HPV45	GTACTAATTTAACATTATGTGCCT
HPV39	GTACCAACTTTACATTATCTACCT
HPV70	GTACTAATTTTACATTGTCTGCCT
P53X	CY5-CCCGC AACCACACAGTCTATGTCTACATATTCTTC GCGGG-DAB
HPV53	AACCACACAGTCTATGTCTACATATAATTC
GAPDHP	TXR-CCCGC CCCAATACGACCAAATCTAAGAGACAAGAGGC GCGGG-BQ2

### LATE-PCR

LATE-PCR was performed in a volume of 40 μl with 5 μl of extracted DNA as template. Each sample was tested in two reactions: A and B. Both reaction mixture contains 1× PCR Buffer (Takara, Japan), 2 mM MgCl_2_ (Promega, USA), 12.5 nM dU plus deoxynucleotide triphosphates (dNTPs, Takara), 37.5 nM limiting primer MY11 (5′-GCMCAGGGWCATAAYAATGG-3′), 500 nM excess primer GP6+ (5′-GAAAAATAAACTGTAAATCATATTC-3′), two units Hot-Start *Taq* polymerase (Takara), and one unit Uracil DNA Glycosylase (UNG, Takara). Probes in reaction A were P6(25 nM), P58 (15 nM ), P68(15 nM), and P59(20 nM), and for reaction B there were P51(25 nM), P66(25 nM), P73(15 nM), P83(15 nM), P45(20 nM) and P53(20 nM). Reaction B also contained internal control primers G100f /R (50 nM) and probe GAPDHP(20nM). All primers and probes were ordered and purchased from Invitrogen Life Technologies (Shanghai, China). A LightCycler480 (Roche, Germany) real time quantative thermo cycler was used for LATE-PCR. The PCR was programmed as below: 10 min at 50°C and 5min at 95°C, followed by 55 cycles (95°C for 15sec, 45°C for 30sec, and 72°C for 30 sec), preceded by a melting program starts at 37°C with 1°C increment per 30 sec interval until 85°C. Fluorescence signals in four different channels were collected and analyzed: FAM (465–510), HEX (533–580), TXR (533–610), and CY5 (618–660). To generate the T_m_ values, melt-curve analysis using the first derivative of fluorescence at temperatures between 37°C and 85°C was used to process the fluorescence data of the probe–target hybridizations.

### Determination of reference value

The 920 HPV-positive samples were further tested using the LPM genotyping assay described above and T_m_ values were collected. The nonparametric rank percentile method determinations of the 2.5th and 97.5th individual percentiles, as well as 95% confidence intervals for the upper and lower limits of each reference interval was used to calculate the reference interval. Results were evaluated using SPSS 16.0 statistical software. The normal distribution was examined by Kolmogorov–Smirnov Z-test.

### Analytical sensitivity and specificity

Recombinant plasmids containing the cloned full-length L1 gene were used to assess the analytical sensitivity of our method.

The inserted DNA fragments of each HPV type were PCR amplified from corresponding HPV positive samples that were diagnosised by the HPV GenoArray test. The pMD19-T vector(Takara) was used as the carrier and the construction of pMD19-T-L1 was performed following the manufacturer’s instruction. The accuracy of cloned L1 fragments were examined by DNA sequencing follow by BLAST, the results were 100% identical to the reference sequences. An ABI3130 genetic analyzer (Applied Biosystems, USA)sequencing instrument was used for sequencing in our experiment.

The concentration of purified plasmids were quantified using a NanoDrop1000 Spectrophotometer (NanoDrop, DE). Copy numbers of the cloned HPV L1 genes in the stock were calculated using the molecular weights of the recombinant plasmids; Quantified plasmids were diluted with TE buffer (pH 8.0) to prepare serial of dilutions with concentration from 1 to 10^6^ copies/ml. Equal amount (40 ng/ml) of human genomic DNA was added into each dilution. Five μl of each plasmid dilution were used as template in LATE-PCR and each HPV type run in 20 replicate reactions per dilution.

To evaluate the specificity of our method, DNA was purified from LBC samples that were diagnosised *Neisseria gonorrhoeae* (ATCC49226)/ *Candida albicans* (ATCC90028)/ *Escherichia Coli* (ATTCC25922)/ *Staphylococcus aureus* (ATTCC25923)/ Herpes simplex virus type II/ *Treponema pallidum*/ *Ureaplasma urealyticum*,/*Mycoplasma hominis* positive using the QIAamp DNA Mini kit. Five microliter of HPV26, 40, 43, 44, 54, 61, 67, 69, 71, and 72 plasmids at a concentration of 10^8^ copies/ml purchased from National Institutes for Food and Drug Control were used as templates in LATE-PCR ().

### Comparison of the HPV GenoArray test with the LPM genotyping assay

We next compared our method with the GenoArray test, 1108 LBC samples were tested by both method in parallel. The GenoArray test is an L1 consensus primer-based PCR assay that covers 21 HPV genotypes, including type16, 18, 31, 33, 35, 39, 45, 51, 52, 56, 58, 59, 68, 53, 66, 6, 11, 42, 43, 44 and 81. The low-risk HPV genotypes 43 and 44 were out of the detection range of the LPM assay and genotypes 70, 73, 82, and 83 could be detected, but not quantified by LPM assay. All GenoArray detection procedures were performed according to the manufacturer’s instructions, and LPM genotyping was conducted as described above. All data were analyzed with SPSS statistical software(version 16.0). The coincidence rate between the two detection methods was assessed using absolute agreement and Cohen’s kappa. The nonparametric McNemar test was used to analyze the complementarity of the two methods and if there was significant difference between the results obtained by the two methods. Difference with *P* value≥0.05 was considered not statistically significant.

### Type-specific sequencing analysis

Sequencing was performed when there was discrepancy in the results between the two assays as described by Peng *et al*. [3]. The PGMY09/11 primer set was used to amplify a specific fragment of the L1 gene from purified DNA. HPV was typed by the sequence of amplified fragments using a type-specific sequencing primer. Sequencing was performed using the BigDye Terminator v3.1 cycle sequencing kit with AmpliTaq DNA polymerase (Applied Biosystems) according to the protocols provided by the manufacturer. The sequences obtained were aligned with reference HPV sequences using the BLAST tool (http://www.ncbi.nlm.nih.gov/BLAST).

## Results

### Reference Tm values of each HPV type

For each HPV type, the results from forty HPV-positive clinical sample were used to establish reference intervals that are with statistically significance. All samples were tested with the LPM assay as described above and the T_m_ values were recorded. Each T_m_ value were normal distribution tested; The reference Tm values were generated and evaluated using SPSS 16.0 software. Reference T_m_ value intervals for each HPV type are shown in Table 2. In separate reactions (A or B), no T_m_ value overlapping was observed.

**Table 2:** T_m_ value of each HPV type.

Reaction	Fluorescent labeling	Excitation and Emission wavelengths (nm)	HPV type	T_m_ Value
A	FAM	465–510	45	49.82–51.00
			16	53.33–55.45
			11	58.00–59.41
			6	64.44–67.22
	HEX	533–580	68	47.09–48.63
			18	51.40–53.00
			33	54.69–55.90
			52	57.11–59.13
			58	61.77–63.89
	CY5	618–660	42	47.49–49.24
			59	52.17–54.13
			35	55.62–56.59
			31	59.93–64.00
B	FAM	465–510	81	50.65–52.10
			56	53.84–55.35
			82	57.03–58.18
			51	59.66–61.20
			66	64.98–66.49
	HEX	533–580	83	57.79–59.75
			73	63.72–65.41
	CY5	618–660	39	45.35–47.35
			70	54.89–56.04
			53	60.46–66.92

### Analytical sensitivity and specificity of the LPM assay

Constructed L1 gene carrying plasmids with gradient concentration (1 to 10^6^ copies/ml) and added human genomic DNA were used as mimic of HPV DNA extracted from clinical sample to assess the sensitivity of LPM assay.

The analytical sensitivity was defined as the lowest concentration (target gene copy number per reaction) that ≥ 95% of test runs produced positive results. The detection limit for HPV6, 11, 16, 18, 33, 35, 45, 52, 56, 58, 66, 68, 73, and 81 was 50 copies/reaction; 500 copies/reaction for HPV31, 42, 51, 53, and 59; and 5 copies/reaction for HPV39, 70, 82, and 83.

### HPV genotyping results of GenoArray test and LPM assay

Both assay detects largely overlapping sets of HPV genotypes, and HPV genotyping results from 1108 LBC samples were compared; the results were mostly concordant or compatible. Out of the 1108 samples tested, four failed to generate melting peak in the control channel of the LPM assay, suggesting that there was either insufficient cells in the collected sample or presence of PCR inhibitor in the extracted DNA. Therefore, results from 1104 samples with used to compare the performance of the two methods. Overall, the absolute agreement between the tests was 98.37% (95% CI: 97.44%–98.97%) and the kappa value was 0.954, indicating good agreement. The positive concordance rate was 98.94% (95% CI: 98.00%–99.44%) and the negative concordance rate was 96.43% (95% CI: 93.35–98.11%) (Table 3). Subsequently, a comparison of the identification of HPV genotypes was made and the results are summarized in Table 4. The two assays were in perfect agreement for the majority of HPV types within the scope, with kappa values of >0.75 except for HPV45, for which the two assays exhibited only moderate agreement (kappa values, 0.40–0.75). More HPV45 positive cases were detected by LPM assay than the by GenoArray, the presence of HPV45 in those samples were further confirmed by sequencing, indicating that the LPM assay was more sensitive in HPV45 detection.

**Table 3:** Comparison of results for the overall detection of HPV DNA by the two assays.

LPM assay result	GenoArray assay result		
	Positive	Negative	Total
Positive	843	9	852
Negative	9	243	252
Total	852	252	1104

**Table 4:** Interassay agreements for individual HPV genotypes detected by two assays.

HPV genotype	No. of samples positive by			Absolute agreement (%)	Kappa value
	LPM	GenoArray/Sequencing	Both		
6	41	41	40	99.82	0.975
11	25	26	24	99.73	0.940
16	141	144	140	99.55	0.980
18	42	43	36	98.82	0.841
31	24	26	24	99.82	0.959
33	29	37	29	99.28	0.875
35	25	26	25	99.91	0.980
39	54	55	53	99.73	0.971
42	20	25	19	99.37	0.841
45	23	9	9	98.73	0.557
51	51	53	50	99.64	0.960
52	161	158	156	99.37	0.974
53	81	81	80	99.82	0.987
56	60	61	59	99.73	0.974
58	103	101	100	99.64	0.978
59	34	41	33	99.18	0.876
66	39	38	38	99.91	0.987
68	14	17	14	99.73	0.902
70	15	14	14	99.91	0.965
73	9	8	8	99.91	0.941
81	60	60	59	99.82	0.982
82	22	20	20	99.82	0.951
83	11	10	10	99.91	0.952

Among the 1104 cases studied, 73 had discordant GenoArray and LPM assay results, six exhibited discordant sequencing and LPM assay for HPV70, 73, 82, and 83. The 73 discordant cases were further analyzed by type-specific PCR followed by sequencing (Table 5). According to the results of sequencing, 40 of them matched the LPM assay results and 31consist with the GenoArray results, the sequencing results disagreed with neither assays in two cases.

**Table 5:** Counts of samples with discordant genotypes reassessed by sequencing.

	Total	Detected by GenoArray	Confirmed by sequencing	Detected by LPM assay	Confirmed by sequencing
6	2	1	0	1	1
11	3	2	1	1	1
16	5	4	2	1	1
18	13	7	5	6	6
31	2	2	2	0	0
33	8	8	6	0	0
35	1	1	1	0	0
39	3	2	2	1	1
42	7	6	1	1	1
45	14	0	0	14	14
51	4	3	1	1	1
52	7	2	0	5	5
53	2	1	1	1	1
56	3	2	2	1	1
58	4	1	1	3	3
59	9	8	7	1	1
66	1	0	0	1	1
68	3	3	1	0	0
81	2	1	0	1	1
70	2	/	/	2	0
73	1	/	/	1	0
82	2	/	/	2	0
83	1	/	/	1	0

Some patients suffered from infection from more than one type of HPV, in the aspect of detecting multiple HPV infection, minor variations were observed between the GenoArray/Sequencing and LPM assay (P≥0.05) (Table 6). Both assays detected multiple infections in about 16.0% of the samples, suggesting promising overall agreement (kappa value = 0.88). Details about multiple infections detected by the two methods are as presented in Table 7, the majority of the mixed HPV infections detected were double infections: 81.1% (146 out of 180) and 79.7% (141 out of 177) by the GenoArray and the LPM assays, respectively. The most complex HPV infection was diagnosis by GenoArray assay, six HPV types were found in single LBC sample.

**Table 6:** The counts of negative, single and multiple HPV infections identified by GenoArray/Sequencing and LPM assay and the consistent or varied results between the two methods (shadded cells).

GenoArray/Sequencing	LPM assay			
	Single HPV type	Multiple HPV type	Negative	Total
Single HPV type	644	23	8	675
Multiple HPV type	22	154	1	177
Negative	6	3	243	252
Total	672	180	252	1104

**Table 7:** Numbers of multiple genotypes identified by GenoArray/Sequencing and LPM assay.

No. of HPV genotypes	GenoArray/Sequencing	LPM
Total	177	180
Two	141	146
Three	32	30
Four	2	2
Five	1	2
Six	1	0

## Discussion

Since the discovery of HPV as the major cause of cervical cancer, various methods have been developed for HPV detection and genotyping. The majority of which are based on molecular biology techniques. The Hybrid Capture 2 system (HC2, Digene Corp., USA) detects 13 HR-HPV types (16, 18, 31, 33, 35, 39, 45, 51, 52, 56, 58, 59, and 68) and five LR-HPV types (6, 11, 42, 43, and 44) by signal amplification technique. It distinguishes high-risk from low-risk types, but incapable of HPV genotyping [11]. However, identifying HPV to genotype is clinically important. International consensus is that persistent infection of HR HPVs such as HPV16, 18, 31, 33, 35, 39, 45, 51, 52, 56, 58, 59, 66, can lead to cervical cancer, while LR viruses infection causes benign cervical tissue alterations or genital warts [12]. Typing HPV infection can provide extra support for disease management and novel primary cervical cancer screening strategies based HPV DNA detection are becoming widely accepted [13]. In addition, new broad-spectrum HPV vaccines are in development [14]. Bivalent (against types 16 and 18), quadrivalent (against types 6, 11, 16, and 18) and nonavalent (against types 6, 11, 16, 18, 31, 33, 45, 52, and 58) vaccines have went through clinical trial [15–21]. Integration of vaccination with screening could reduce not only cervical cancer onset but also risks associated with over-treatment.

Currently, HPV GenoArray test is one of the most used methods for HPV genotyping in China. This method covers 21 HPV genotypes including 6, 11, 16, 18, 31, 33, 35, 39, 42, 43, 44, 45, 51, 52, 53, 56, 58, 59, 66, 68 and 81. Numerous studies have proven its reproducibility and clinical sensitivity. Therefore, we used it as reference for the LPM assay in this study [8, 22]. However, we found the drawbacks of GenoArray are being prone to contamination and its time-consuming procedure [8]. In addition, it is sometimes difficult to give an unambiguous result for samples with low viral load, because the determination of results is solely based on direct visualization.

Real-time PCR has long been used in Vitro pathogen detection and identification, its limitation is only limited number of targets can be detected in one reaction, due to the limited fluorescence channel provided by the real-time PCR instrument [23–26]. However, it is often desirable to detect and identify multiple types of pathogen that potentially present in a clinical sample. To addressing this problem, molecular beacons labeled with different fluorophores that specific to certain target sequences are introduced to simultaneously detect and identify multiple targets in a single reaction [27]. Chakravorty *et al*. performed rapid universal identification of bacterial pathogens from a list of 111 species in 64 different genera using a molecular beacon melting temperature signature technique [28]. Additionally, El-Hajj *et al*. described a molecular beacon based system that successively distinguished 27 different Mycobacterium species [29]. These assays identify multiple targets by fluorescence signals in combination with the melting temperatures of the hybridized probe-target dimmer. Based on the above technique, we developed a new HPV detection and genotyping system—the LPM assay, and its performance was validated on clinical samples by comparing with the GenoArray test. In HPV typing, excellent agreement (kappa = 0.954; 95% CI, 97.44%–98.97%) between the two assays was observed, which was in line with data previously reported comparison between another assay based on LATE-PCR and lateral flow and the GenoArray test [30]. The sensitivity, specificity and accuracy of the LPM assay were 98.94%, 96.43% and 98.37%, respectively. Suggests HPV genotypes can be successfully identified by the LPM assay with high accuracy. For individual HPV genotype, the GenoArray and LPM assays were in near-perfect agreement, the majority of targeted HPV types are with kappa values >0.75 except for HPV45, where the two assays had moderate agreement (kappa values, 0.40–0.75) and the LPM assay showed better sensitivity to HPV 45 than GenoArray. Of the 1104 LBC samples tested with the LPM assay, only 33 of them had disagreement with GenoArray or type-specific sequencing. These results indicate that the LPM method can successfully detect HPV infections and identify HPV genotypes with high accuracy. Additional practical advantages with the LPM assay are that it costs less (less than 1.5 US dollars per sample) and is faster (<2 h) than the GenoArray assay; On the other hand, because in our assay the LATE-PCR and fluorescence measurement is conducted in sealed tubes, carryover contamination is less likely to happen.

In conclusion, in our study, the LPM assay showed similar clinical performance for genotyping as the HPV GenoArray test and seemed to be a promising alternative approach for HPV testing. It is sensitive, economical and high throughput that could be used for rapid and specific detection of 23 high- and low-risk HPV genotypes.
